# Deformation mechanisms to ameliorate the mechanical properties of novel TRIP/TWIP Co-Cr-Mo-(Cu) ultrafine eutectic alloys

**DOI:** 10.1038/srep39959

**Published:** 2017-01-09

**Authors:** J. T. Kim, S. H. Hong, H. J. Park, Y. S. Kim, J. Y. Suh, J. K. Lee, J. M. Park, T. Maity, J. Eckert, K. B. Kim

**Affiliations:** 1 Hybrid Materials Center (HMC), Faculty of Nanotechnology and Advanced Materials Engineering, Sejong University, 209 Neugdong-ro, Gwangjin-gu, Seoul 143-747, Republic of Korea; 2 High Temperature Energy Materials Research Center, Korea Institute of Science and Technology, Hwarangno 14-gil 5, Seoungbuk-gu, Seoul 136-791, Republic of Korea; 3 Division of Advanced Materials Engineering, Kongju National University, Cheonan 330-717, Republic of Korea; 4 Global Technology Center, Samsung Electronics Co., Ltd, 129 Samsung-ro, Yeongtong-gu, Suwon-si, Gyeonggi-do 443-742, Republic of Korea; 5 Department Materials Physics, Montanuniversität Leoben, Jahnstraße 12, A-8700 Leoben, Austria; 6 Erich Schmid Institute of Materials Science, Austrian Academy of Sciences, Jahnstraße 12, A-8700 Leoben, Austria

## Abstract

In the present study, the microstructural evolution and the modulation of the mechanical properties have been investigated for a Co-Cr-Mo (CCM) ternary eutectic alloy by addition of a small amount of copper (0.5 and 1 at.%). The microstructural observations reveal a distinct dissimilarity in the eutectic structure such as a broken lamellar structure and a well-aligned lamellar structure and an increasing volume fraction of Co lamellae as increasing amount of copper addition. This microstructural evolution leads to improved plasticity from 1% to 10% without the typical tradeoff between the overall strength and compressive plasticity. Moreover, investigation of the fractured samples indicates that the CCMCu alloy exhibits higher plastic deformability and combinatorial mechanisms for improved plastic behavior. The improved plasticity of CCMCu alloys originates from several deformation mechanisms; i) slip, ii) deformation twinning, iii) strain-induced transformation and iv) shear banding. These results reveal that the mechanical properties of eutectic alloys in the Co-Cr-Mo system can be ameliorated by micro-alloying such as Cu addition.

The development of nano- or ultrafine-structured materials (NSMs or USMs) is an important issue of materials research due to their outstanding high strength compared to coarse-grained counterparts, as indicated by the well-known Hall-Petch relationship, and a large number of NSMs and USMs have been developed in Ti-, Fe-, Zr-, Cu-, Ni- and Al-based alloys^1^,^2^,^3^,^4^,^5^,^6^. Moreover, eutectic alloys also have been evaluated as one of the key fields of materials research due to their high strength and straightforward process for fabrication of NSMs or USMs. In particular, the prime advantages of eutectic alloys are their high castability originating from single melting behavior, their low melting temperature, the ease of manufacturing ultrafine microstructures and a high possibility to use them in a multitude of engineering applications^5^,^6^. However, typical ultrafine-eutectic alloys (UEAs) exhibit poor plasticity because these materials are deformed mainly by highly localized shear bands and thus show catastrophic failure following highly limited plasticity similar to NSMs or USMs^7^,^8^,^9^. These restrictions such as limited ductility and low deformability etc. render them unsuitable for engineering applications^1^,^2^,^3^,^4^. Various countermeasures for improving ductility have been reported for eutectic alloys via controlling the propagation of shear bands through introducing a bimodal (or multi-modal) microstructure^3^,^4^,^7^,^10^,^11^,^12^,^13^,^14^,^15^. For instance, ultrafine eutectic composites with homogeneously embedded primary dendritic phase dispersions have been reported for a variety of Ti-1,16, Fe-4,10 and Al-based^14^ alloy systems, which present a superior combination of both high strength and improved plasticity. Systematic investigations on the deformation mechanisms of these ultrafine eutectic-dendrite composites demonstrated that the deformation proceeds preferentially through dislocation-based mechanisms in the micro-scale primary dendrite phases and then shear bands are generated in the ultrafine eutectic matrix^1^,^4^,^10^,^14^,^16^. In order to prevent the catastrophic failure induced from the lack of mechanisms for controlling the shear bands, these micro-scale toughening phases, e.g. primary dendrite phases, are essential in facilitating plastic deformation via controlling the shear bands through a generation of multiple shear bands and blocking/deflecting their propagation. However, despite the thus achievable plasticity improvement, a decrease of strength is inevitable^1^,^4^,^14^. More recently, microstructural tailoring of UEAs without primary toughening phases such as in the case of duplexed microstructural (structural and/or chemical) eutectic alloys has been proposed in Ti-11,17, Al-3 and Fe-based^12^ UEAs. The detailed deformation mechanisms responsible for the improvement of plasticity without tradeoff between strength and plasticity have been interpreted by the evolved morphology of eutectic colonies, which can lead to the formation of a large number of shear bands^2^,^4^,^7^,^10^, their rotation motion with wavy propagation of shear bands^11^,^12^,^13^.

In recent years, there have been studies concerning the microscopic deformation mechanisms of ultrafine eutectic alloys using the indentation size effect (ISE)^18^,^19^. The ISE is a well-known phenomenon, which has been found in a variety of materials e.g. metals, ceramics, and composites, indicating a change of the indentation hardness with the size of the indents^19^. In order to describe the ISE behavior, the traditional Meyer’s law can be used, which gives an expression considering the maximum load and contact depth of an indent^20^,


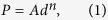


where the exponent *n* is the Meyer index, *P* is the maximum load, *d* is the contact depth and *A* is a constant. For the normal ISE mode, the value of Meyer’s index (*n*) is represented by *n* < 2, whereas *n* > 2 is relevant for the reverse ISE mode. In addition, when *n* < 2, i.e. normal ISE, this phenomenon is believed to be correlated to geometrically necessary dislocations (GNDs). Therefore, it can be suggested that the deformation of materials exhibiting a lower *n* value is more dominated by dislocations than that for higher *n* values, which is responsible for high hardness at low loading conditions^19^,^20^. Along with this line, Das *et al*. have reported that the dislocation-based deformation mechanisms including dislocation-lamellae interaction and slip transfer across the lamellae interfaces are still important factors for improving the global plasticity as well as for controlling shear bands in Ti-Fe-(Sn) UEAs^18^,^19^. However, the deformation behavior of dislocation-based deformation mechanisms, as well as the interaction between deformation bands and lamellar interfaces in UEAs have not yet been studied systematically in other eutectic systems. Moreover, Co-based alloys have been recognized as dental and biomedical materials for use as dental implants, surgical tools or artificial hip joints, etc. due to their excellent strength, corrosion resistance, biological inertness and wear resistance^21^,^22^,^23^. Especially, the Co-Cr-Mo alloy system is one of the commonly used alloy systems in the medical field and has also a high potential for other engineering applications because of their good mechanical properties. So far, however, there have been only a few reports on the formation and microscopic deformation behavior of Co-Cr-Mo UEAs. Therefore, it is the objective of the present work to investigate the mechanical properties and the deformation behavior of Co-Cr-Mo UEAs improved by a small amount of Cu addition, and we focus on the interaction of the lamellar interface and dislocation-based deformation mechanisms such as slip or twinning etc.

In this study, the microscopic deformation mechanisms of Co-Cr-Mo-(Cu) UEAs have been investigated. The effect of Cu addition on the mechanical properties and the deformation behavior has been evaluated using the indentation size effect and transmission electron microscopy analysis obtained from interrupted compression tests. The microscopic deformation mechanisms will be systemically discussed, revealing that the mechanical properties of eutectic alloys can be improved by the multiple mechanisms involved in the TWIP, TRIP, and lamellar interfacial interaction.

## Results

**Figure 1:**
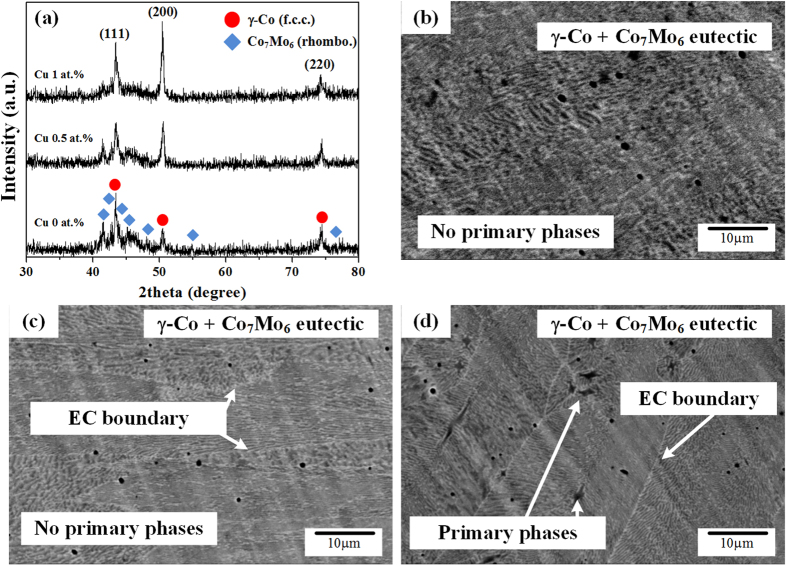
(**a**) XRD patterns and (**b**–**d**) SEM images obtained from the as-cast Co_65−x_Cr_13_Mo_22_Cu_x_ alloys with *x* = 0, 0.5 and 1 at.%.

The XRD patterns of the Co_65−x_Cr_13_Mo_22_Cu_x_ with x = 0, 0.5 and 1 at.% alloys (CCMCs) are very similar: all alloys are comprised of the f.c.c. γ-Co (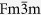) and rhombohedral Co_7_Mo_6_ 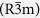 phases, as shown in Fig. 1(a). As the Cu content increases from the Co-Cr-Mo ternary eutectic alloy (CCM), the peak intensity corresponding to the γ-Co phase becomes stronger and the peak intensity of the Co_7_Mo_6_ phase decreases along with the finding that the width of the reflections becomes broader, indicating microstructural refinement. This suggests that the addition of Cu leads to an increased volume fraction of γ-Co and refined Co_7_Mo_6_ without change of the constituent phases observed for the Co-Mo-Cr ternary eutectic alloy. Figure 1(b–d) show characteristic SEM back-scattered electron (BSE) micrographs of the present alloys, confirming the presence of an ultrafine microstructure constituted two phases (γ-Co and Co_7_Mo_6_). Figure 1(b) shows the microstructure of the CCM, revealing a typical ultrafine eutectic structure without primary dendrites. The microstructure of the Cu 0.5 at.% alloy (CCMC0.5) is displayed in Fig. 1(c). The CCMC0.5 alloy also shows a typical ultrafine eutectic structure without primary dendrite phases, whereas the Cu 1 at.% alloy (CCMC1) exhibits small-sized primary phase(s) verified as f.c.c. γ-Co in the eutectic colonies. From image analysis, the volume fraction and size of the primary phase(s) in the CCMC1 alloy are estimated to be less than 1 vol.% and 3 μm, respectively.

**Figure 2:**
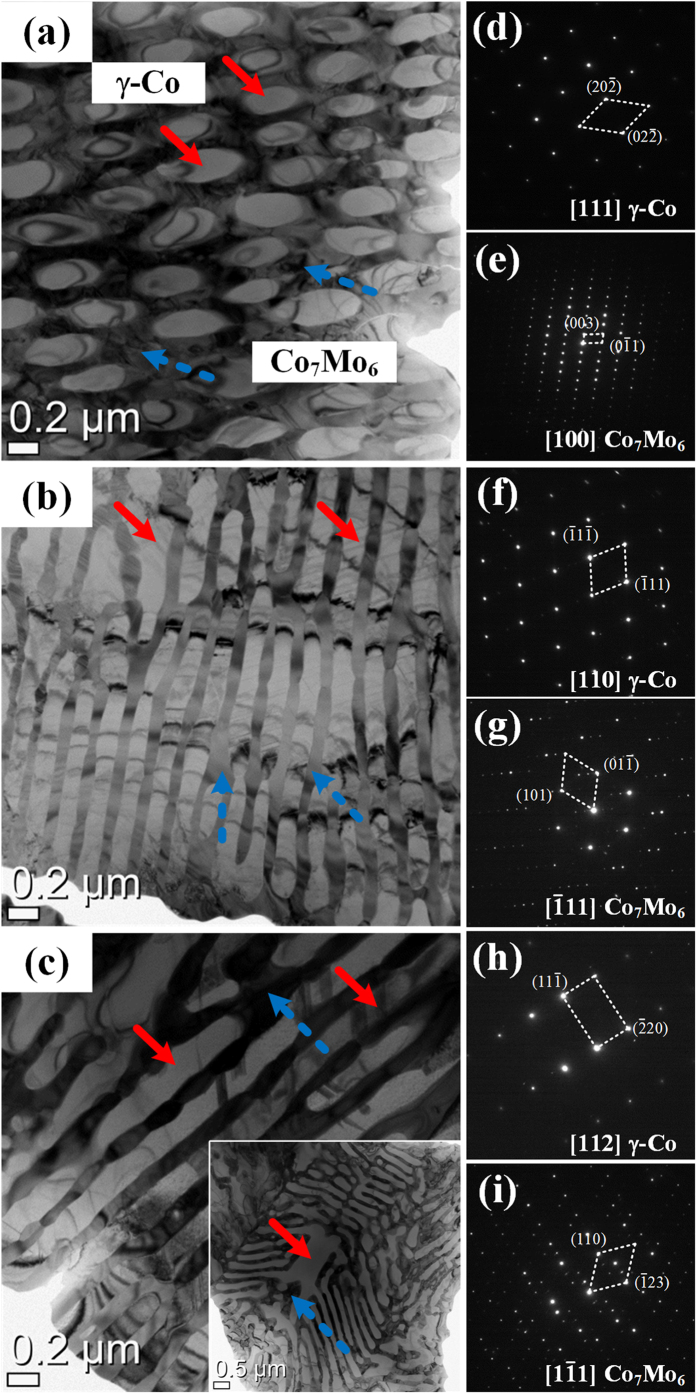
TEM bright-field (BF) images and elected area electron diffraction (SAED) patterns obtained from the as-cast Co_65−x_Cr_13_Mo_22_Cu_x_ alloys with x = 0, 0.5 and 1 at.%: (**a**) BF image, (**d–e**) SAED patterns of x = 0 at.%, (**b**) BF image, (**f–g**) SAED patterns of x = 0.5 at.% and (**c**) BF image, and (**h–i**) SAED patterns of x = 1 at.%.

TEM bright field (BF) images and selected area electron diffraction (SAED) patterns of Co_65−x_Cr_13_Mo_22_Cu_x_ with x = 0, 0.5 and 1 at.% alloys are displayed in Fig. 2. EDX analysis indicates that the present alloys are comprised of alternating brighter contrast areas that correspond to Co-rich phases (marked by red arrows) and darker contrast areas that correspond to Co_7_Mo_6_ phases (marked by blue dotted arrows). Moreover, the microstructural observation of the CCM alloy clearly demonstrates a broken lamellar structure appearing as small Co-rich islands, as shown Fig. 2(a). The SAED patterns obtained from discrete Co-rich phases are identified to correspond to the [111] zone axis of the f.c.c. γ-Co phase and those of the dark contrast areas are verified as the [100] zone axis of the rhombohedral Co_7_Mo_6_ phase. On the other hand, the CCMC0.5 and CCMC1, exhibit drastically changed microstructures compared to the CCM, showing a typical alternating lamellar eutectic microstructure (Fig. 2(b and c)). The SAED patterns obtained from the CCMC0.5 are identified as the [110] zone axis of γ-Co and the [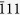] zone axis of Co_7_Mo_6_, as shown in Fig. 2(f and g). Moreover, as demonstrated by Fig. 2(c,h and i), the CCMC1 exhibits a typical alternating ultrafine eutectic structure with primary phase in the eutectic colony as already deduced above from the SEM results and the [112] zone axis of γ-Co and the [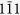] zone axis of the Co_7_Mo_6_ phase are identified by SAED pattern analysis. The volume fractions of γ-Co measured from more than 10 TEM images is verified to be 50.3 vol.% (CCM), 62.1 vol.% (CCMC0.5) and 67.5 vol.% (CCMC1) and the average lamellar spacing obtained from 10 BF images is found to decrease with increasing Cu content from 0.17 ± 0.05 μm for x = 0.5 to 0.1 ± 0.03 μm for x = 1, respectively. The microstructural features, i.e. the volume fraction (V_f of Co_) and the average lamellar spacing (λ) are summarized in Table 1.

**Figure 3:**
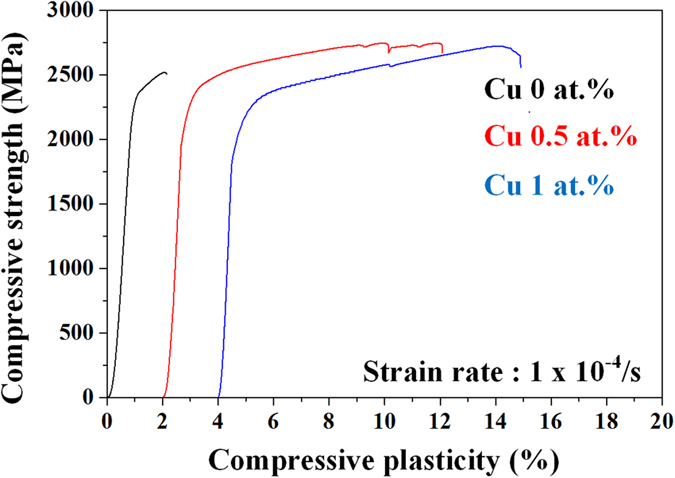
Engineering compressive stress-strain curves of the as-cast Co_65−x_Cr_13_Mo_22_Cu_x_ alloys with *x* = 0, 0.5 and 1 at.%.

Figure 3 presents the compressive engineering stress-strain curves of Co_65−x_Cr_13_Mo_22_Cu_x_ with x = 0, 0.5 and 1 at.% alloys at room temperature, which were determined at a strain rate of 1 × 10^−4^/s. The CCM exhibits a high yield strength (σ_y_) of about 2.3 GPa with a limited plasticity of less than 1.5%, whereas the CCMC0.5 and CCMC1 show considerably improved plasticity up to ~10.5% without the typical tradeoff between strength and plasticity. At the same time, the points of drop of stress (pop-in) in compressive stress-strain curves are observed which indicate crack initiation and their blockage at the interface. Thus, it indicates a good resistance to crack propagation^16^. The values of yield strength (σ_y_), fracture strength (σ_f_) and plastic strain (ε_p_) obtained from the present alloys are summarized in Table 1.

**Figure 4:**
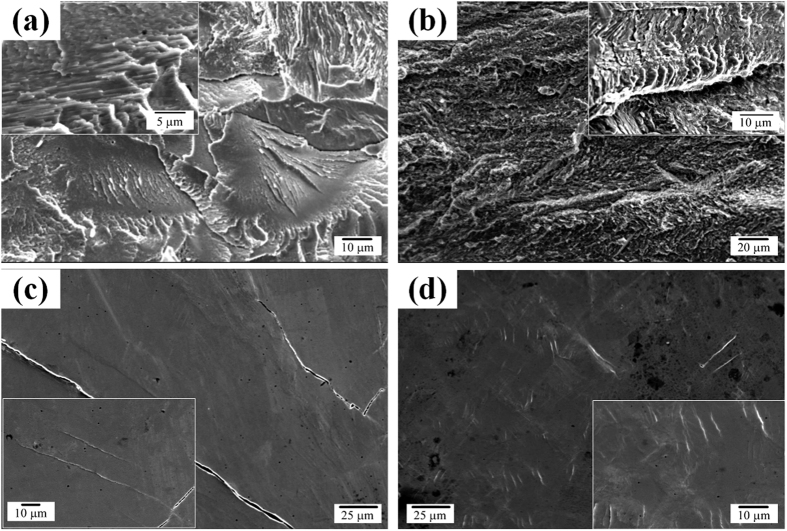
SEM secondary electron images of fracture/lateral surface obtained from the fractured Co_65−x_Cr_13_Mo_22_Cu_x_ alloys with *x* = 0 and 0.5 at.% alloys: (**a**) fracture surface and (**c**) lateral surface of x = 0 at.%, (**b**) fracture surface and (**d**) lateral surface of x = 0.5 at.%.

To understand the deformation mechanisms with the exception of the primary phase effect, the deformed and fractured CCM and CCMC0.5, verified to have a fully eutectic microstructure, were further investigated in more detail. Figure 4 shows SEM images of the fracture and lateral surface obtained from fractured specimens. The fracture and lateral surface of the CCM, as depicted in Fig. 4(a and c), exhibit typical cleavage patterns and a small amount of deformation traces, e.g. shear bands. This indicates the absence of mechanisms for control of propagating deformation bands. On the other hand, the CCMC0.5 shows a more complicated fracture surface and a lot of shear bands. Moreover, these shear bands exhibit a short propagating path, which means that the propagation of shear bands is effectively impeded.

In general, Meyer’s law has been widely used to describe the existence of ISE for materials such as ceramics and metals^19^,^20^. It has been mentioned above that Meyer’s law is expressed as *P* = *A x d*^*n*^. Especially, the exponent *n*, referred to as Meyer index, is usually considered as a decision criterion for ISE. Hence, using logarithmic transformation, the *n* value has been estimated from the slope of ln *P*–ln *d* plots, according to the modified Eq. (1) in the following way:


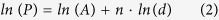


**Figure 5:**
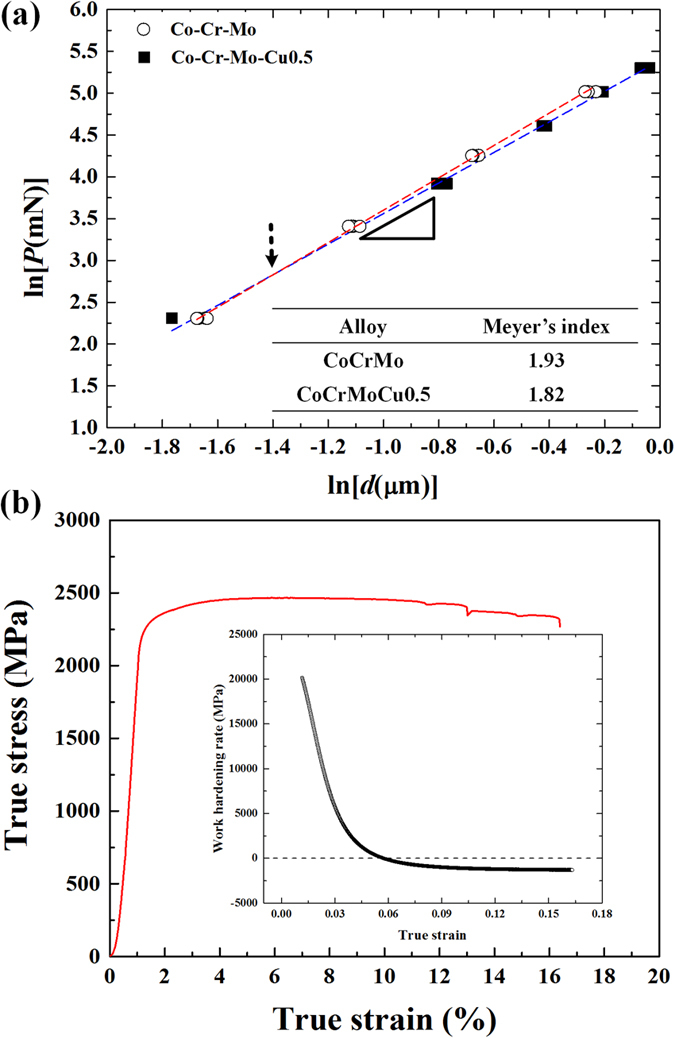
(**a**) log-log plots between maximum load and contact depth obtained from CCM and CCMC0.5 alloys and calculated Meyer indexes and (**b**) true stress-strain curve and work hardening rate of CCMC0.5 alloy.

Figure 5(a) exhibits the ln *P* − ln *d* plots of maximum load and contact depth obtained from the CCM and CCMC0.5. The Meyer index, corresponding to the slope of the fitted lines, decreases upon Cu addition from 1.93 to 1.82, pointing to a normal ISE behavior in the present alloys. The smaller *n* value indicates that the size effect becomes more dominant and the strain gradient increases due to the formation of a large population of sessile dislocations^19^. Moreover, in the lower maximum load condition, the fitted lines intersect, as marked by a black arrow in Fig. 5. This indicates a stronger hardening behavior of the CCMC0.5 induced by the formation of GNDs associated with the strain gradient in the initial stage of plastic deformation^19^,^20^,^24^,^25^.

Figure 5(b) reveals the true stress-strain curve and work hardening rate obtained from CCMC0.5 alloy. The work hardening rate is estimated by Hollomon equation defined as S = Kε^n^ where S, K and ε are the true stress, strength coefficient and true strain, respectively. The high work hardening rate was found at the initial stage of plastic deformation which can link with the higher strain gradient of ISE behaviors as shown in Fig. 5(a). Moreover, as processing of plastic deformation, the work hardening rate is gradually decreased and then after the middle of plastic deformation state, the work hardening rate reveals the negative values, which correspond with work softening behavior^26^. It is strongly indicated that the dominant deformation mechanisms are altered as the deformation stage.

From the Taylor dislocation model^27^,^28^, the stress of f.c.c. metals, σ, is controlled by the Taylor stress and is described by:


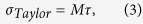


where *M* is the Taylor factor which acts as an isotropic interpretation of the crystalline anisotropy at continuum level, and τ is the shear flow stress. The shear flow stress, τ, is related to the dislocation density, *ρ*, by:


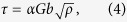


where *G* is the shear modulus, *b* is the magnitude of the Burgers vector, and *α* is an empirical factor depending on the dislocation structure. Therefore, the dislocation density, *ρ*, is determined from Eqs (3, 4) as:


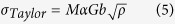


To a first approximation, and assuming that the deformation occurs preferentially at the γ-Co lamellae, it is an essential prerequisite that the Taylor factor, *M*, is reported as 3.06 for f.c.c. metals^29^ and the empirical factor, α, is around 0.3^30^. Moreover, the burgers vector can also be considered to be very similar for the CCM and CCMC0.5 due to the identical crystal structures. For this reason, Eq. (5) can be simplified as:


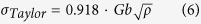


The dislocation density, *ρ*, is can be expressed by:


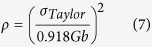


With the Taylor stress and the shear modulus obtained from equal strain level in true stress-strain curves and nanoindentation, the estimated dislocation densities according to Eq. (7) are 2.9 × 10^14^ m^−2^ and 4.4 × 10^14^ m^−2^ for CCM and CCMC0.5, respectively. The estimated dislocation density is a function of indentation depth and a linear superposition of the statistically stored dislocation (SSD) density with the GNDs density 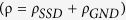. Although, the estimated value of the dislocation density includes the density of SSDs, the density of SSDs is proportional to the hardness of the material^30^ and the average hardness of CCM obtained from varying the load is higher than that of the CCMC0.5, which indicates that the density of SSDs in CCM is higher than for CCMC0.5. Therefore, even when the density of SSDs is excluded, the dislocation density of CCMC0.5 is still much higher than that of CCM, which denotes that the deformation of CCMC0.5 is more dominantly influenced by dislocations at the earlier deformation stage.

**Figure 6:**
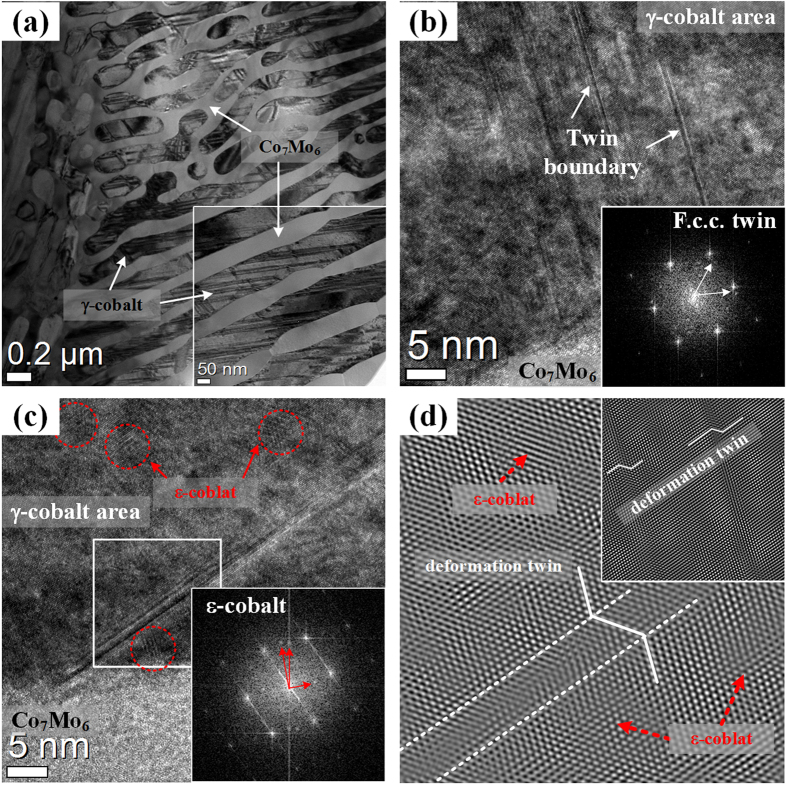
TEM analysis obtained from deformed CCMC0.5 specimen up to 3%: (**a**) BF image displaying deformation trace in the γ-Co lamellae, (**b–c**) high resolution (HR) images and Fast fourier transform (FFT) patterns of γ-Co lamellae showing the deformation twins and transformed ε-Co phases and (**d**) inverse FFT filtered images clearly exhibiting deformation twins corresponding to the white square area of (**c**).

Figure 6 displays TEM images obtained from CCMC0.5 specimens deformed up to ~3%. Slip bands are found at the γ-Co lamellae, as shown in Fig. 5(a). The high magnification image and the fast Fourier transformation (FFT) pattern, shown in Fig. 6(b and c), reveal the formation of deformation twins and ε-Co phase at the γ-Co lamellae, which are generated by a strain-induced phase transformation (SIPT)^31^,^32^,^33^. These ε-Co precipitates are located near the deformation twins situated at the γ-Co lamellae. The inverse FFT filtered image, obtained from the white squared area in Fig. 6(c), clearly reveals the existence of deformation twins and ε-Co phase.

**Figure 7:**
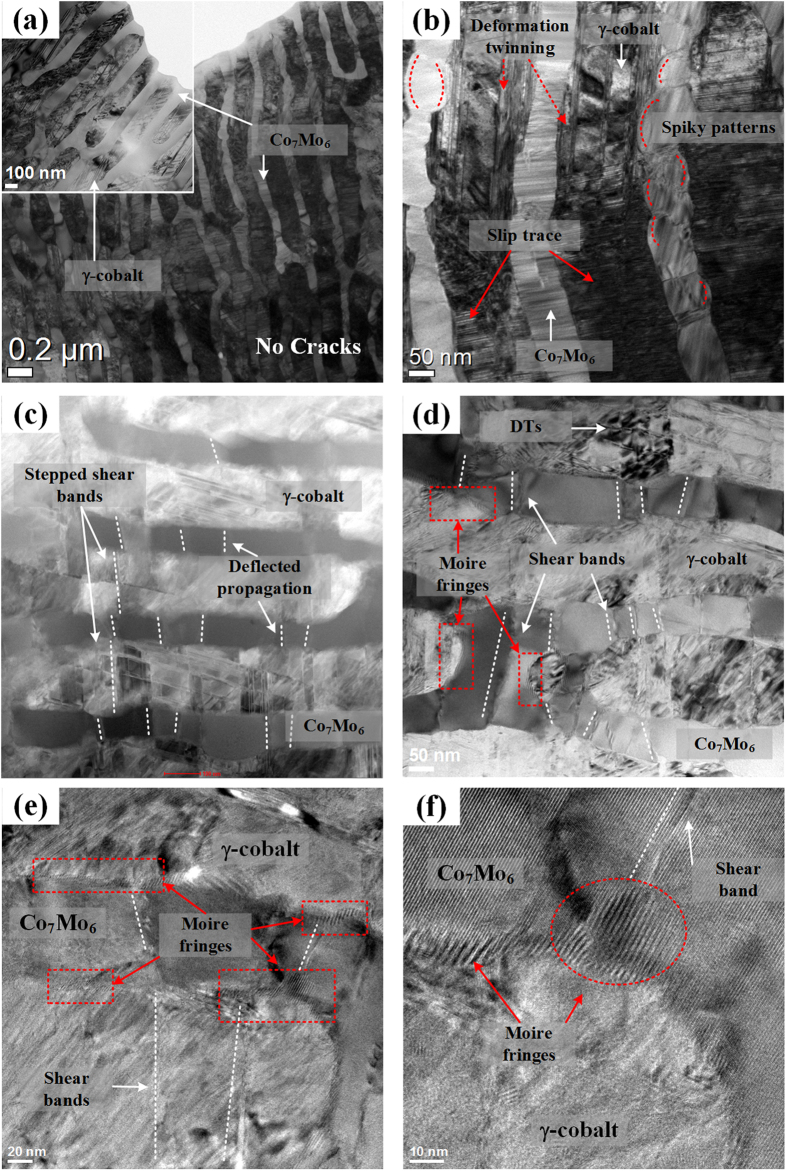
TEM analysis obtained from fractured CCMC0.5 specimen: (**a**,**b**) BF images showing the severely deformed lamellar structure, (**c,d**) high magnified STEM and BF images clearly showing shear bands and (**e,f**) HR images showing the interaction at the lamellar interface.

TEM bright field (BF) and high-resolution (HR) images of a fractured CCMC0.5 specimen are shown in Fig. 7. Observation at low magnification reveals that a high density of deformation bands, giving evidence for dislocation-based deformation, are generated in both γ-Co and Co_7_Mo_6_ without crack generation at the lamellar interface, as shown in Fig. 7(a). Moreover, the high magnification BF image in Fig. 7(b) exhibits severely deformed γ-Co lamellae, showing a large amount of slip traces and deformation twinning. Furthermore, spiky morphologies are frequently found at the lamellar interface, as marked by dotted red lines. Figure 7(c and d) display the presence of shear bands passing through the ultrafine lamellar interfaces. These shear bands are deflected in their propagation at the lamellar interface, which indicates the interaction between shear bands and lamellar interface. Moreover, Moiré fringes are observed at the lamellar interface and the sharp edges of the spiky patterns are found at the tip of the shear band. The more highly magnified images [Fig. 7(e and f)] clearly reveal that the shear bands are deflected at the lamellar interface and heavy Moiré fringes are located near the tip of the shear bands. This indicates that the lamellar interfaces in the CCMC0.5 effectively accommodate the applied stress and then impede the propagation of deformation bands which involved in fracture immediately. More details about all figures are given in the supplementary information.

## Discussion

XRD analysis suggests that the constituent phases do not change when adding a small amount of Cu to the CCM. The refined Co_7_Mo_6_ phase and the increased volume fraction of γ-Co phase induced by Cu addition indicates that Cu addition leads to a variation of the compositions towards the Co-rich area within/near the fully eutectic zone. The CCM and CCMC0.5 represent the fully eutectic zone and the CCMC1 corresponds to the near fully eutectic zone. The alterations in the features of the lamellae such as the observed transition from a broken lamellar to a well-aligned lamellar structure and the change of the volume fraction are evidence for the compositional variations. Based on the combination of TEM observation and UTM analysis, one can deduce that the well-aligned lamellar eutectic microstructure induced by a small amount of Cu addition shows a higher possibility of plastic deformation. Moreover, notwithstanding that the volume fraction of the soft solid solution phase, e.g. γ-Co, is increased, an unremarkable decrease of the yield strength is found, which can be correlated with the refined microstructure and a large amount of lamellar interface induced by the Cu addition. Furthermore, a lower Meyer index value is linked with the formation of GNDs to create a higher strain gradient in the early stage of deformation. Generally, in the normal ISE mode, a smaller *n* value corresponds to a higher strain gradient during the plastic deformation, which implicates that a higher number of dislocations is activated. The estimated dislocation density also indicates a larger number of GNDs in CCMC0.5, which is associated with a higher strain gradient. Therefore, dislocation pile-ups or dislocation networks may have formed at the lamellar interfaces and can act as pinning sites leading to the formation of a large population of sessile dislocations, which increases the strain gradient^19^,^20^,^24^,^25^. Thus, the higher hardening behavior and the refined microstructure are correlated with the interrupted decrease of the yield strength.

The fracture and lateral surfaces obtained from fractured CCM, CCMC0.5 indicate that the fracture of the CCM has occurred by a few shear bands and/or cracks, which can be extrapolated from the lateral observation. The typical cleavage patterns observed at the fracture surface suggest that the fracture was effectuated in a brittle manner. These findings, i.e. the cleavage fracture morphologies and smaller deformation traces, indicate the absence of mechanisms for the avoidance of precipitous fracture. On the other hand, the CCMC0.5 displays abundant deformation traces and a large number of shear bands at the fracture surface and the lateral surface of the failed specimen. Moreover, the observed shear bands on the lateral surface show a much shorter propagation path than in the case of the CCM. This suggests a variety deformation bands and/or mechanisms for controlling the deformation behavior and the propagation of shear bands provoking failure can be effectively restrained. These findings can explain the higher plastic deformability before fracture, and it can be inferred that the deformation initiates via dislocation movement and proceeds through shear banding.

A detailed analysis of the deformation behavior of the CCMC0.5 was performed by TEM investigations of 3% plastically deformed and fractured specimens, as shown in Figs 6 and 7, respectively. In the earlier state of plastic deformation (Fig. 6) dislocation movement-induced slip traces and deformation twins are generated at the γ-Co lamellae, which indicates that the γ-Co lamellae are preferentially deformed in comparison with the lamellae of the Co_7_Mo_6_ intermetallic compound. The high magnification TEM images and the FFT patterns reveal areas of deformation twins together with phase transformed areas from f.c.c. γ-Co to h.c.p. ε-Co near the deformation twins. The inverse FFT images clearly display the deformation twins in the γ-Co lamellae. Generally, a strain-induced γ → ε phase transformation is commonly observed for polycrystalline cobalt alloys and γ-Co with low stacking fault energy facilitates twinning upon deformation^33^. Deformation twins have been also found in the various types of f.c.c. structural materials with low stacking fault energy^34^. Therefore, it can be indirectly inferred that the small amount of Cu addition to the CCM ternary eutectic alloy can lead to a decrease of the stacking fault energy of the γ-Co phase, which means that deformation twinning and SIPT are preferred to other deformation mechanisms. The applied stresses, therefore, are accommodated by the formation of deformation twins and phase transformations at the early stage of deformation^35^. Generally, deformation twinning is preferentially occurred by the motion of Shockley partial dislocations at a critical strain level^23^. With continuously increasing strain, the ε-Co phase nucleates gradually near the twin boundaries. Internal stresses and external applied stresses develop near the interfaces in deformed materials, which has been reported to influence the deformation and fracture behavior^23^,^36^. Therefore, this indicates that the SIPT is promoted by concentrated external applied and residual stresses near the twin boundaries. Moreover, deformation twinning may play a crucial role in reaching high strength due to the phenomenon that twin boundaries act as pinning sites for dislocation movement^37^. As mentioned above, deformation twin boundaries and ε-Co particles nucleated by SIMT are well-known pinning sites for dislocation motion. Since most of the dislocations locate in the γ-Co lamellae in the early deformation stage, deformation twin boundaries and ε-Co particles can act as effective obstacles to impede the movement of dislocations, which leads to a higher strain gradient associated with a smaller *n* value, as demonstrated by Fig. 5. Moreover, the higher work hardening rate of earlier deformation state is also related with increased pinning site such as deformation twins and ε-Co particles, which dislocation slip is blocked, thereby reducing the dislocation mean free path through a dynamic Hall-Petch effect^35^. Thus, the CCMC0.5 with a higher volume fraction of soft Co phase shows a similar yield strength as the CCM. Furthermore, both deformation twinning and SIPT may contribute to improving plastic deformability via the well-known TWIP (twinning-induced plasticity) or TRIP (transformation-induced plasticity) effects, which noted that both of these effects may provide strengthening^34^,^38^,^39^,^40^. These deformation characteristics are exceptional mechanisms in ultrafine eutectic alloys and can be linked to the unique strain accommodating behavior during deformation of ultrafine eutectic alloys. In the latter stage of deformation, shear bands are generated before the alloy finally fractures with improved plasticity. As shown in Fig. 7, no cracks are observed at the lamellar interfaces and boundaries after fracture. This finding corresponds to the mechanically stable lamellar interface and higher strain concentrations are found at the whole lamellar areas. Usually, stress concentrations induced by the pile-up at the interfaces/boundaries are relieved by either slip or cracking in the adjacent phases. The easy slip transfer leads to global plasticity, which adversity of crack activation. Otherwise, when the slip transfer becomes more difficult than crack activation due to problems such as a large mismatch of crystallographic features and mechanical mismatch, the crack can be initiated before slip transfer, which corresponds to cleavage fracture behavior^4^,^18^,^41^,^42^. Therefore, it can be inferred that the stress concentration at the γ-Co lamellae promotes slip transfer before initiation of cracks in the Co_7_Mo_6_ lamellae related to the catastrophic failure. Hence, the partial plastic deformation is enacted by dislocation-based deformation mechanisms (slip, TWIP and TRIP) and then shear bands are activated in the higher energy state without cracking, as shown in Fig. 7(c and d).

**Figure 8:**
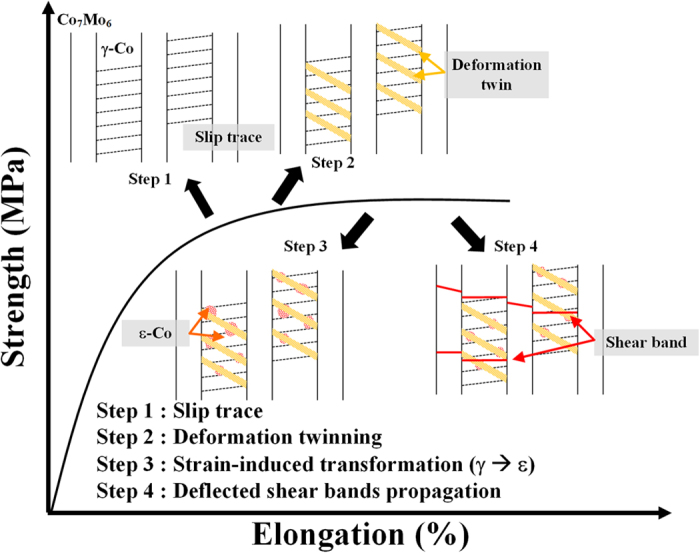
Schematic showing the evolved deformation mechanisms of CCMC0.5 alloy as the 4 steps: slip, deformation twinning, strain-induced phase transformation and interaction between the shear band and lamellar interface.

Once the stress level reaches a higher state, shear bands are initiated and propagate across the lamellar structure. The shear band is the one of the well-known mechanisms, which leads to the work softening behavior. As shown in Fig. 5(b), the later stage of deformation, the CCMC0.5 reveals the negative work softening rate which means the shear bands become the more dominant deformation mechanism than other dislocation-based mechanisms. When these shear bands traverse a lamellar interface, they undergo deflection and/or blocking, thus illustrating the effect of lamellar interfaces for preventing premature failure. Especially, the spiky patterns found at the γ-Co side of the lamellar interface correspond to the vestige of sluggishly propagating shear bands, which demonstrates that the propagation of shear bands is interrupted at the γ-Co lamellae during cutting and passing through the lamellar structure. During such shear band propagation, some of the deformation twin boundaries and TRIP- or TWIP-induced hardened γ-Co lamellae are destroyed, which means the disturbing the propagation of shear bands, and is the reason for the sluggish propagation behavior. The interaction between shear bands and deformed γ-Co lamellae can lead to the accommodation of shear strain and cause delayed propagation of shear bands^43^,^44^,^45^. Moreover, Moiré fringes are discovered at the lamellar interfaces, which are the result from the strain-induced overlapping of lamellae. The Moiré fringes also able to demonstrate the localized deformation and the high density of defects such as dislocations^46^. Similar to nanostructured composites^47^,^48^,^49^, the Moiré fringes are caused by mechanical mismatch, e.g. elastic modulus, and plastic strain, between the neighboring phases formed to accommodate the shear strain. In other words, Moiré fringes generated by the individual interface deformation mode originating from coherent interfaces in order to accommodate the applied stress are widely developed within severely deformed alloys^3^,^10^. Furthermore, the sharp edge of the spiky patterns located near the tip of the shear bands is verified as heavy Moiré fringes, which substantiates considerable interaction between the shear bands and the lamellar interface as strong evidences for the accommodation of shear strain at the lamellar interface during deformation. Therefore, in the CCMC0.5, the lamellar interfaces play a central role in the containment of catastrophic failure via mechanisms accommodating the external stress by means of deformed individual lamellar interfaces and impede the propagation of shear bands. Hence, as described in the above discussion, the consumption of applied stress relevant to plastic deformability is mediated by i) slip in the γ-Co lamellae, ii) deformation twinning, iii) strain-induced phase transformation from γ-Co to ε-Co, and iv) suppressing the propagation of shear bands by individual lamellar interfaces. The 4 different steps of plastic deformation are schematically illustrated in Fig. 8. These findings reveal that it is possible to develop superior mechanical properties in ultrafine eutectic alloys through understanding the micromechanical behavior involved in the slip, TWIP, TRIP and interfacial lamellar interaction with shear bands.

## Conclusions

In conclusion, we summarize the microstructure, mechanical properties, and the deformation behavior of Co-Cr-Mo-(Cu) eutectic alloys based on the major findings of the present research focusing on the effect of minor element addition.

- The small amount of Cu addition (0.5 and 1 at.%) to the Co-Cr-Mo ternary eutectic alloy leads to the evolution of the eutectic structure from a broken lamellar to the well-aligned lamellar structure without alteration of the constituent phases but with the varied volume fraction of γ-Co phase induced by the additional small amount of Cu.
- From TEM analysis on the deformed/fractured CCMC0.5, the initial plastic deformation of the CCMC0.5 alloy with continuously well-aligned lamellar structure and decreased Meyer index (*n*) associated with higher strain gradient pointing to the formation of geometrically necessary dislocations is triggered by dislocation slip, deformation twinning, and strain-induced phase transformation in the γ-Co lamellae.
- In the higher strain state, shear bands involved in fracture are generated and propagate across the lamellar structure. However, when the shear bands pass through the lamellar structure, they undergo deflection and/or blocking, which induces interaction between the lamellar interface and the shear bands, illuminating the effect of lamellar interfaces for preventing premature failure.

These novel findings of a unique strain accommodation behavior in ultrafine eutectic alloys can give valuable information for better understanding the superior mechanical properties of ultrafine eutectic alloys.

## Methods

The Co_65−x_Cr_13_Mo_22_Cu_x_ (x = 0, 0.5 and 1 at.%) alloys were prepared by arc-melting of Co, Cr, Mo and Cu of 99.99% purity under Ar atmosphere on a water-cooled Cu hearth. Cylindrical rods with 2 mm diameter and 50 mm length were fabricated using suction casting into a water-cooled Cu mold. Phase analysis was carried out by X-ray diffraction (XRD, Rigaku-D/MAX-2500/PC) with Cu Kα_1_ radiation (λ = 1.5406 Å). Structural analysis was performed using scanning electron microscopy (SEM, JEOL JSM-6390) and transmission electron microscopy (TEM, Tecnai-F20). Thin foil samples for TEM analysis were prepared by conventional ion milling (Gatan, Model 600) and focused ion beam thinning (FIB, Helios D-456). The mechanical properties at room temperature were evaluated under uniaxial compression loading with an initial strain rate of 10^−3^/s. For compression testing, cylindrical specimens with a 2:1 aspect ratio were prepared from the as-cast rods. Indentation tests were performed in a load-control mode conducted with a different maximum loading condition from 10 mN to 200 mN using a Nanoindenter (CSM, NHT-X). The hardness and the elastic modulus were derived using the Oliver-Pharr method^50^.

## Additional Information

**How to cite this article**: Kim, J. T. *et al*. Deformation mechanisms to ameliorate the mechanical properties of novel TRIP/TWIP Co-Cr-Mo-(Cu) ultrafine eutectic alloys. *Sci. Rep.* **7**, 39959; doi: 10.1038/srep39959 (2017).

**Publisher's note:** Springer Nature remains neutral with regard to jurisdictional claims in published maps and institutional affiliations.

## Supplementary Material

Supplementary Information

## Figures and Tables

**Table 1:** Microstructural features and compressive mechanical properties of the as-cast Co_65−x_Cr_13_Mo_22_Cu_x_ alloys with *x* = 0, 0.5 and 1 at.%.

Alloys	Microstructure		Mechanical properties		
	V_f_ of γ-Co (%)	Average lamellar spacing (μm)	Yield strength (MPa)	Fracture strength (MPa)	Plastic strain (%)
CCM	50.3 ± 0.7	0.17 ± 0.05	2306.88 ± 3	2522.69 ± 3	1.31 ± 0.4
CCMC0.5	62.1 ± 0.8	0.11 ± 0.03	2300.30 ± 4	2753.96 ± 5	9.23 ± 0.6
CCMC1	67.5 ± 1.2	0.1 ± 0.02	2156.65 ± 5	2730.18 ± 6	10.05 ± 0.5
