# Supplementary material for: Deformation mechanisms to ameliorate the mechanical properties of novel TRIP/TWIP Co-Cr-Mo-(Cu) ultrafine eutectic alloys
Source: Sci Rep. 2017 Jan 9;7:39959. doi: 10.1038/srep39959 (PMC5220307; doi:10.1038/srep39959)
Supplement: Supplementary Information [file srep39959-s1.pdf]

Supplementary information

Deformation mechanisms to ameliorate the mechanical properties of novel TRIP / TWIP Co-Cr-Mo-(Cu) ultrafine eutectic alloys

J.T. Kim[1], S.H. Hong[1], H.J. Park[1], Y. S. Kim[1], J.Y. Suh[2], J. K. Lee[3], J.M. Park[4] , T. Maity[5], J. Eckert[5,6] and K.B. Kim[1]

[1]Hybrid Materials Center (HMC), Faculty of Nanotechnology and Advanced Materials Engineering, Sejong University, 209 Neugdong-ro, Gwangjin-gu, Seoul 143-747, Republic of Korea

[2]High Temperature Energy Materials Research Center, Korea Institute of Science and Technology, Hwarangno 14-gil 5, Seoungbuk-gu, Seoul 136-791, Republic of Korea

[3]Division of Advanced Materials Engineering, Kongju National University, Cheonan 330-717, Republic of Korea

[4]Global Technology Center, Samsung Electronics Co., Ltd, 129 Samsung-ro, Yeongtong-gu, Suwon-si, Gyeonggi-do 443-742, Republic of Korea

[5]Department Materials Physics, Montanuniversität Leoben, Jahnstraße 12, A-8700 Leoben, Austria

[6]Erich Schmid Institute of Materials Science, Austrian Academy of Sciences, Jahnstraße 12, A-8700 Leoben, Austria

Abstract

In the present data, the microstructural evolution and the modulation of the mechanical properties have been investigated for a Co-Cr-Mo (CCM) ternary eutectic alloy by addition of a small amount of copper (0.5 and 1 at.%). The microstructural data reveal a distinct dissimilarity in the eutectic structure such as a broken lamellar structure and a well-aligned lamellar structure, and an increasing volume fraction of Co lamellae as increasing amount of copper addition. This microstructural evolution leads to improved plasticity from 1 % to 10 % without the typical tradeoff between the overall strength and compressive plasticity. Moreover, data of the fractured samples indicates that the CCMCu alloy exhibits higher plastic deformability and combinatorial mechanisms for improved plastic behavior. The improved plasticity of CCMCu alloys originates from several deformation mechanisms; i) slip, ii) deformation twinning, iii) strain-induced transformation and iv) shear banding. These results reveal that the mechanical properties of eutectic alloys in the Co-Cr-Mo system can be ameliorated by micro-alloying such as Cu addition.

Value of the data

- **The novel findings in these data offer the new possibilities of deformation mechanisms for ultrafine eutectic alloys**
- **It could be a high potential concerning the various possibilities of improving mechanical properties**
- **To be compared to the previous data, it can serve the novel approach strategy as a benchmark for other researchers**
- **As providing the novel approach, the potential of specific research field can be improved through more diverse approaches with various possibilities**

Data

This date article includes phase analysis (XRD), microstructure (SEM, TEM), mechanical properties (UTM), fractured specimen (SEM, TEM), and Meyer's index graph (graph). The evolution of phase and microstructure as small amount of Cu contents are exhibited by XRD, SEM, and TEM data. By the change of phase and microstructural features, the mechanical properties are dramatically improved as shown by UTM. TEM data of deformed / fractured specimens suggest that the small addition of Cu can lead to change of deformation mechanisms which identified by Meyer index (n) associated with higher strain gradient. Moreover, the TRIP / TWIP effects with interfacial interaction plays a crucial role for improving mechanical properties even in ultrafine eutectic alloys. These data reveal that it is possible to develop superior mechanical properties in ultrafine eutectic alloys through understanding the micromechanical behavior involved in slip, TWIP, TRIP and interfacial lamellar interaction with shear bands.

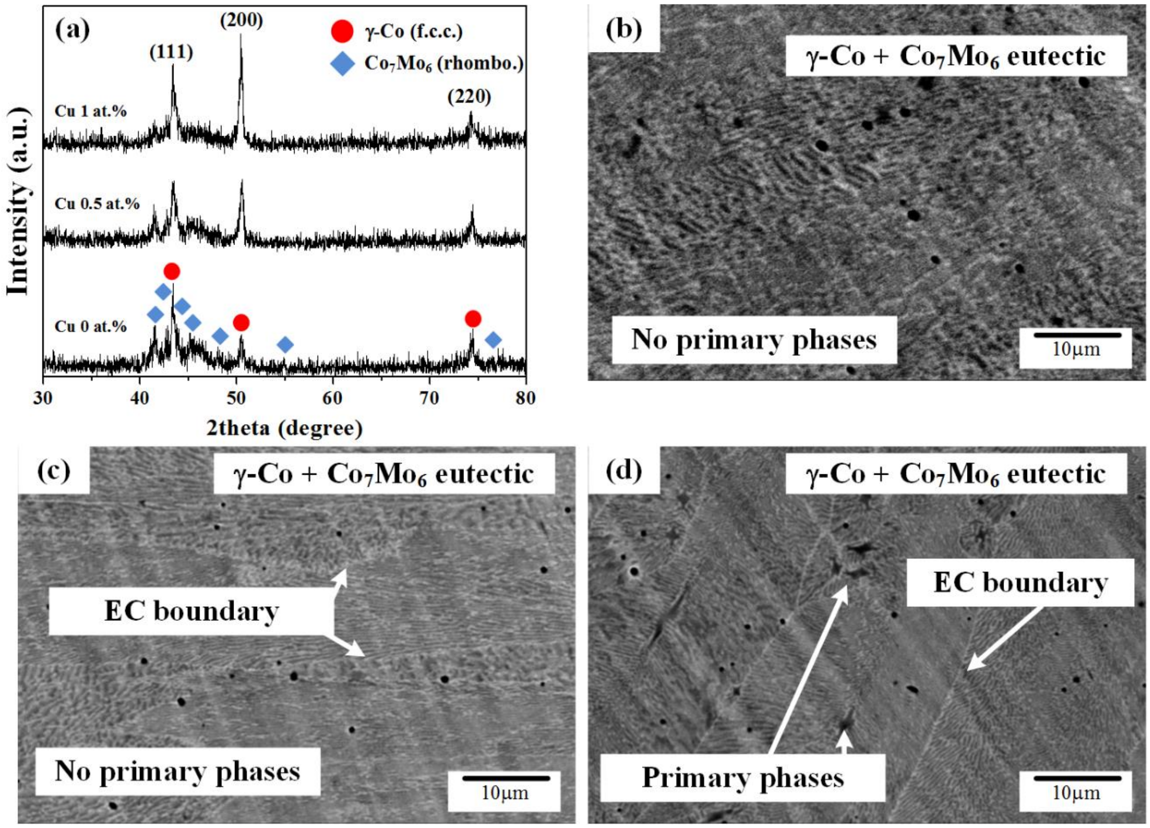

The XRD patterns of the $Co_{65-x}Cr_{13}Mo_{22}Cu_x$ with x = 0, 0.5 and 1 at.% alloys (CCMCs) are very similar: all alloys are comprised of the f.c.c. γ-Co and rhombohedral Co_7Mo_6 phases, as shown in supplementary Fig. 1 (a). As the Cu content increases from the Co-Cr-Mo ternary eutectic alloy (CCM), the peak intensity corresponding to the γ-Co phase becomes stronger and the peak intensity of the Co_7Mo_6 phase decreases along with the finding that the width of the reflections becomes broader, indicating microstructural refinement. This suggests that the addition of Cu leads to an increased volume fraction of γ–Co and refined Co7Mo6 without change of the constituent phases observed for the Co-Mo-Cr ternary eutectic alloy. The supplementary Figs. 1 (b)-(d) show characteristic SEM back scattered electron (BSE) micrographs of the as-cast $Co_{65-x}Cr_{13}Mo_{22}Cu_x$ specimens with x = 0, 0.5 and 1 at.%, confirming the presence of an ultrafine microstructure constituted two phases (γ-Co and Co_7Mo_6). The supplementary

Fig. 1 (b) shows the microstructure of the Cu-free alloy (CCM), revealing a typical ultrafine eutectic structure without primary dendrites. The microstructure of the Cu 0.5 at.% alloy (CCMC0.5) is displayed in The supplementary Fig. 1 (c). The CCMC0.5 alloy also shows a typical ultrafine eutectic structure without primary dendrite phases, whereas the Cu 1 at.% alloy (CCMC1) exhibits small sized primary phase(s) verified as f.c.c. γ-Co in the eutectic colonies. From image analysis, the volume fraction and size

of the primary phase(s) in the CCMC1 alloy are estimated to be less than 1 vol.% and 3 m, respectively.

TEM bright field (BF) images and selected area electron diffraction (SAED) patterns of $Co_{65-x}Cr_{13}Mo_{22}Cu_x$ with x = 0, 0.5 and 1 at.% alloys (CCMCs) are displayed in the supplementary Fig. 2. EDX analysis indicates that the present alloys are comprised of alternating brighter contrast areas that correspond to Co-rich phases (marked by red arrows) and darker contrast areas that correspond to Co_7Mo_6

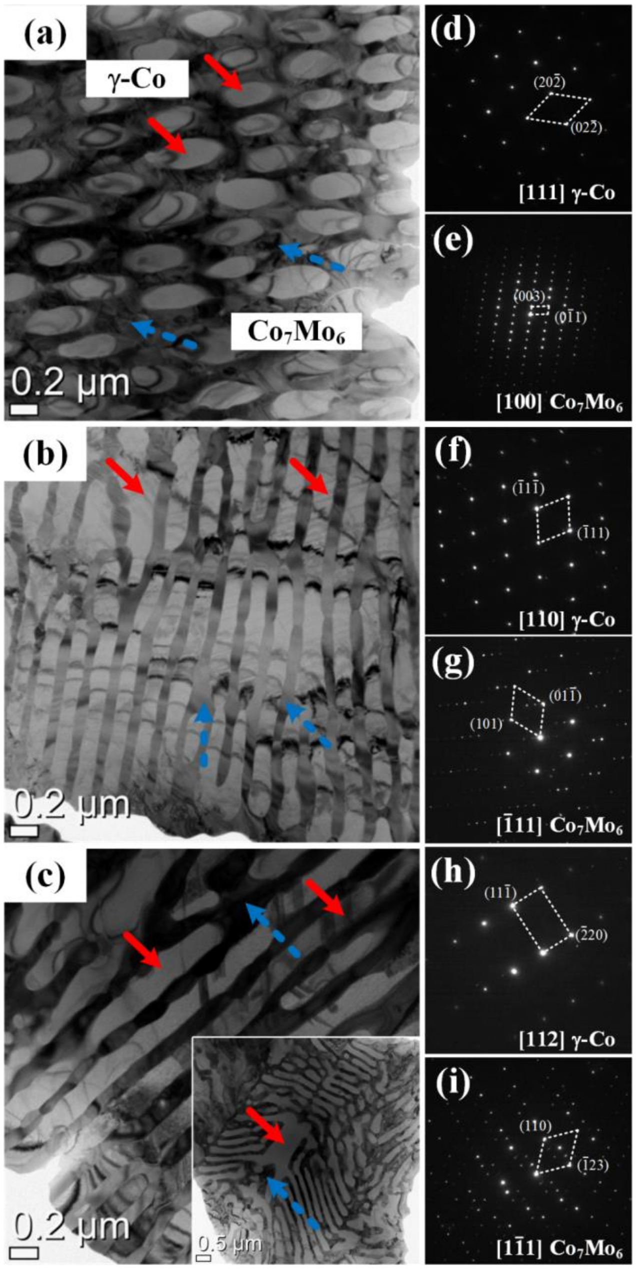

phases (marked by blue dotted arrows). Moreover, the microstructural observation of the CCM alloy clearly demonstrates a broken lamellar structure appearing as small Co-rich islands, as shown the supplementary Fig. 2 (a). The SAED patterns obtained from discrete Co-rich phases are identified to correspond to the [111] zone axis of the f.c.c. -Co phase and those of the dark contrast areas are verified as the [100] zone axis of the rhombohedral Co_7Mo_6 phase. On the other hand, the Cu-containing alloys, e.g. CCMC0.5 and CCMC1, exhibit drastically changed microstructures compared to the CCM alloy, showing a typical alternating lamellar eutectic microstructure (supplementary Figs. 2 (b) and (c)). The SAED patterns obtained from the CCMC0.5 alloy are identified as the [110] zone axis of -Co and the $[\bar{1}11]$ zone axis of Co_7Mo_6, as shown in supplementary Figs. 2 (f) and (g). Moreover, as demonstrated by supplementary Figs. 2 (c), (h) and (i), the CCMC1 alloy exhibits a typical alternating ultrafine eutectic structure with primary phase in the eutectic colony as already deduced above from the SEM results and the [112] zone axis of -Co and the $[11\bar{1}]$ zone axis of the Co_7Mo_6 phase are identified by SAED pattern analysis. The volume fractions of -Co measured from more than 10 TEM images is verified to be 50.3 vol.% (CCM), 62.1 vol.% (CCMC0.5) and 67.5 vol.% (CCMC1) and the average lamellar spacing obtained from 10 BF images is found to decrease with increasing Cu content from 0.17 ± 0.05 m for x = 0 0.5 to 0.1 ± 0.03 m for x = 1, respectively. The microstructural features, i.e. the volume fraction (Vf of Co) and the average lamellar spacing ( ) are summarized in supplementary Table 1.

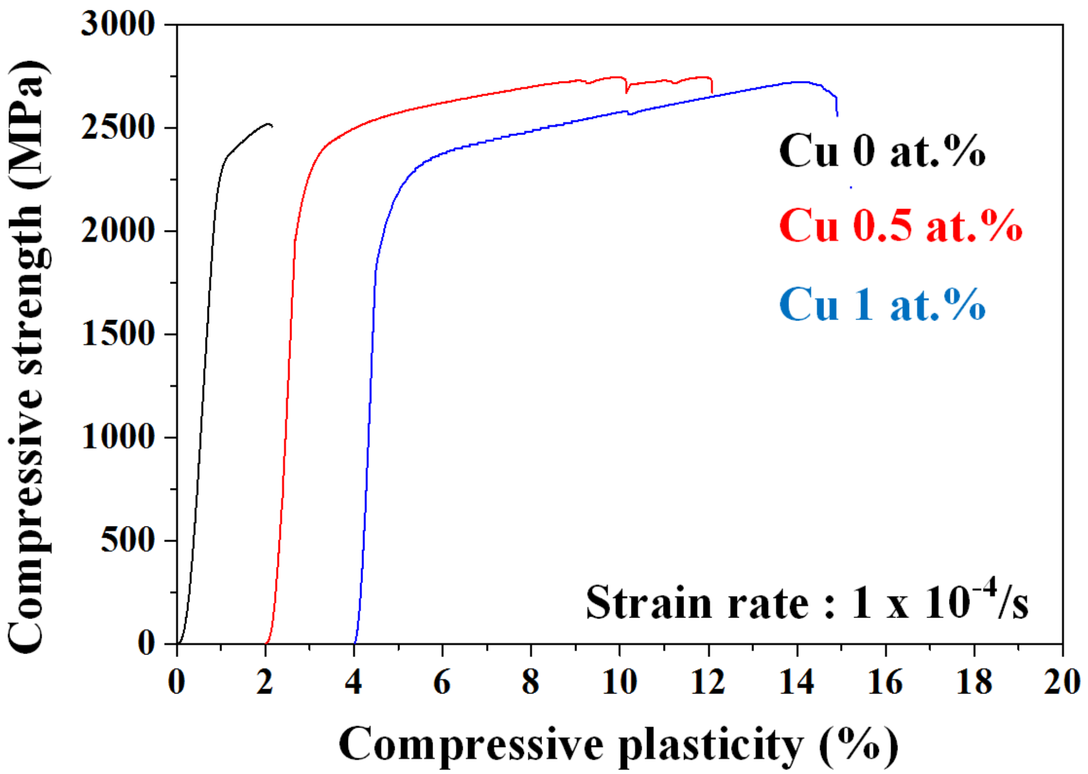

The supplementary Fig. 3 presents the compressive engineering stress-strain curves of $Co_{65-x}Cr_{13}Mo_{22}Cu_x$ with x = 0, 0.5 and 1 at.% alloys (CCMCs) at room temperature, which were determined at a strain rate of 1 x 10^{-4}/s. The Cu-free alloy (CCM) exhibits a high yield strength ($_y$) of about 2.3 GPa with a limited plasticity of less than 1.5%, whereas the Cu-containing alloys (CCMC0.5 and CCMC1) show considerably improved plasticity up to ~10.5% without the typical tradeoff between strength and plasticity. The values of yield strength (σ_y), fracture strength (σ_f) and plastic strain (ε_p) obtained from the present alloys are summarized in supplementary Table 1.

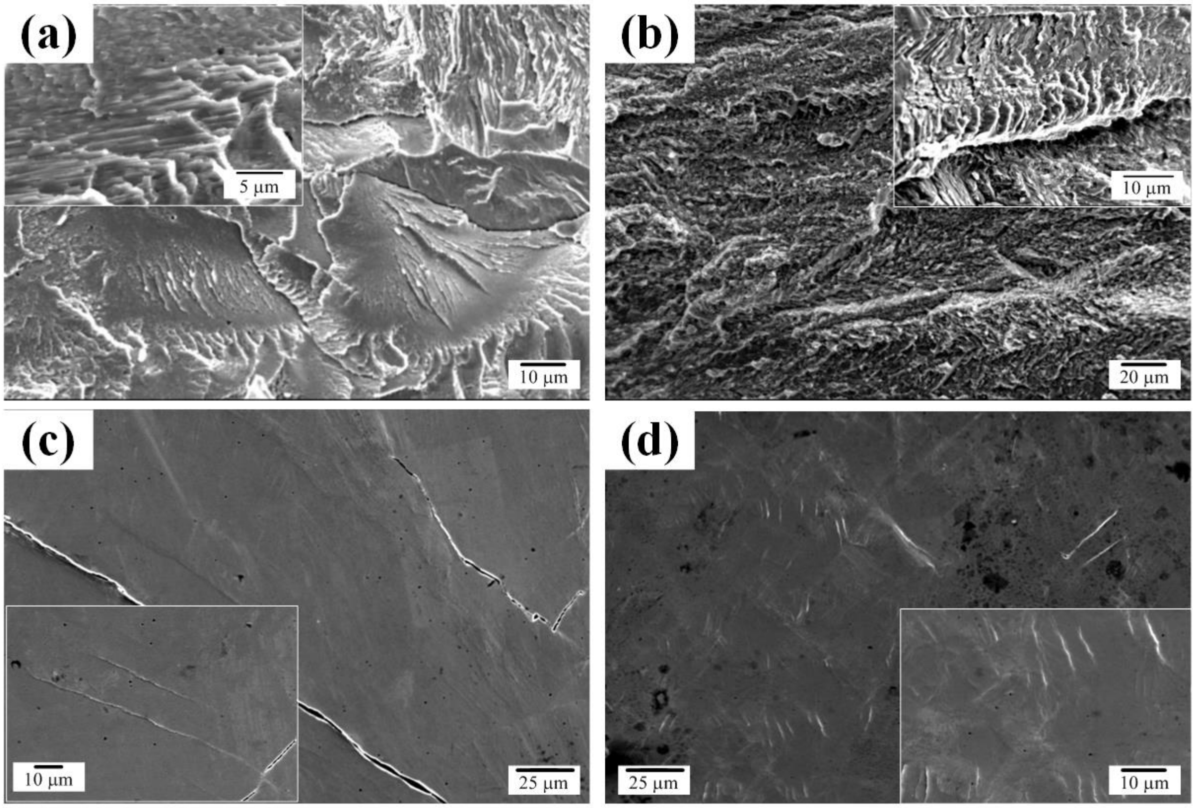

To understand the deformation mechanisms with the exception of the primary phase effect, the deformed and fractured CCM and CCMC0.5 alloys, verified to have a fully eutectic microstructure, were further investigated in more detail. The supplementary Fig. 4 shows SEM images of the fracture surface and the lateral surface obtained from fractured specimens. The fracture surface and the lateral surface of the CCM alloy, as depicted in supplementary Figs. 4 (a) and (c), exhibit typical cleavage patterns and a small amount of deformation traces, e.g. shear bands. This indicates the absence of mechanisms for control of propagating deformation bands. On the other hand, the CCMC0.5 alloy shows a more complicated fracture surface and a large amount of shear bands. Moreover, these shear bands exhibit a short propagating path, which means that the propagation of shear bands is effectively impeded. Therefore, it can be suggested that the small amount of Cu addition can provide a variety deformation mechanisms for controlling the fracture behavior in CCMC eutectic alloys.

In general, Meyer's law has been widely used to describe the existence of ISE for materials such as ceramics and metals. It has been mentioned above that Meyer's law is expressed as P = A x dn (1).

Especially, the exponent n, referred to as Meyer index, is usually considered as a decision criterion for ISE. Hence, using logarithmic transformation, the n value has been estimated from the slope of ln P – ln d plots, according to the modified Eq. (1) in the following way:

$$\ln (P)=\ln (A)+n\cdot\ln(d) \qquad (2)$$

The supplementary Fig. 5 exhibits the ln P – ln d plots of maximum load and contact depth obtained from the CCM and CCMC0.5 alloys. The Meyer index, corresponding to the slope of the fitted lines, decreases upon Cu addition from 1.93 to 1.82, pointing to a normal ISE behavior in the present alloys. The smaller n value indicates that the size effect becomes more dominant and the strain gradient increases due to the formation of a large population of sessile dislocations (see in Ref. 1). Moreover, in the lower maximum load condition, the fitted lines intersect, as marked by a black arrow in supplementary Fig. 5. This indicates a stronger hardening behavior of the CCMC0.5 alloy induced by the formation of geometrically necessary dislocations (GNDs) associated with the strain gradient in the initial stage of plastic deformation (See in Ref. 1, 2, 3, and 4).

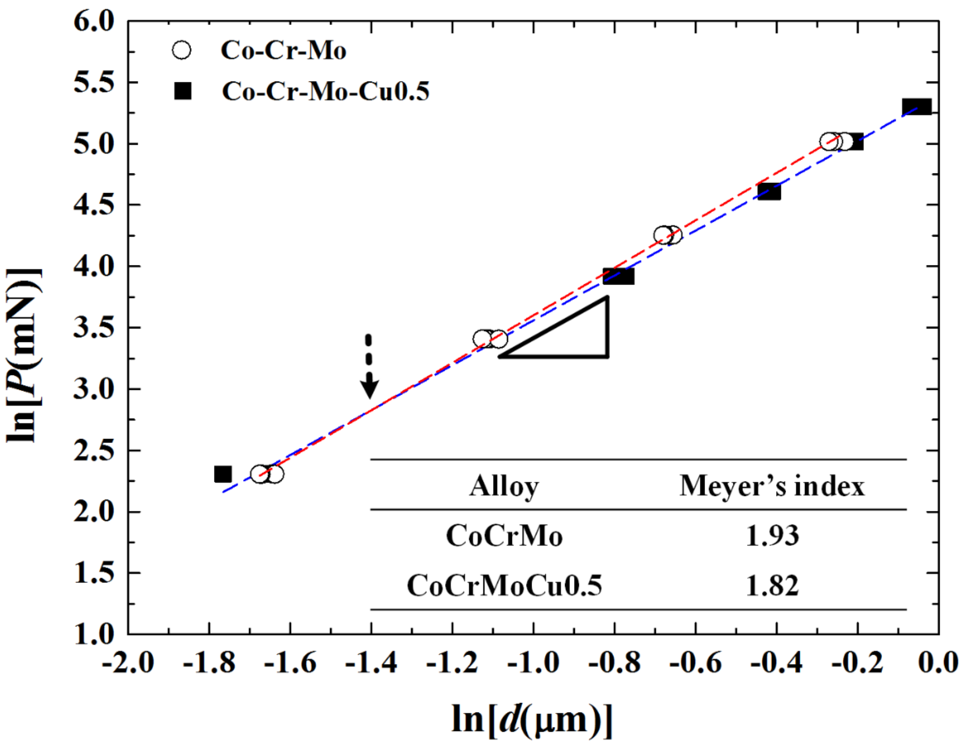

From the Taylor dislocation model shown in Ref. 5 and 6, the stress of f.c.c. metals, σ, is controlled by the Taylor stress and is described by:

$$\sigma_{Taylor}=M\tau \quad , (3)$$

where M is the Taylor factor which acts as an isotropic interpretation of the crystalline anisotropy at continuum level, and τ is the shear flow stress. The shear flow stress, , is related to the dislocation density, ρ, by:

$$\tau=\alpha Gb\sqrt{\rho} \quad , (4)$$

where G is the shear modulus, b is the magnitude of the Burgers vector, and α is an empirical factor depending on the dislocation structure. Therefore, the dislocation density, ρ, is determined from Eqs. (3)-(4) as:

$$\sigma\_Taylor=M\alpha Gb\sqrt{\rho} \quad (5)$$

To a first approximation, and assuming that the deformation occurs preferentially at the γ-Co lamellae, it is an essential prerequisite that the Taylor factor, M, is reported as 3.06 for f.c.c. metals (see in Ref. 7) and the empirical factor, α, is around 0.3 (see in Ref 8). Moreover, the burgers vector can also be considered to be very similar for the CCM and CCMC0.5 alloys due to the identical crystal structures. For this reason, Eq. (5) can be simplified as:

$$\sigma_{Taylor}=0.918\cdot Gb\sqrt{\rho} \quad (5\text{-}1)$$

The dislocation density, , is can be expressed by:

$$\rho=(\sigma_{Taylor}/0.918Gb)\text{^}2 \quad (5\text{-}2)$$

With the Taylor stress and the shear modulus obtained from equal strain level in true stress-strain curves and nanoindenation, the estimated dislocation densities according to Eq. (5-2) are 2.9 x10^{14} m^{-2} and 4.4 x 10^{14} m^{-2} for CCM and CCMC0.5, respectively. The estimated dislocation density is a function of indentation depth and a linear superposition of the statistically stored dislocation (SSD) density with the geometrically necessary dislocation (GND) density ($\rho= \rho_{SSD}+\rho_{GND}$). Although, the estimated value of the dislocation density includes the density of SSDs, the density of SSDs is proportional to the hardness of

the material (see in Ref. 8) and the average hardness of CCM obtained from varying the load is higher than that of the CCMC0.5 alloy, which indicates that the density of SSDs in CCM is higher than for CCMC0.5. Therefore, even when the density of SSDs is excluded, the dislocation density of CCMC0.5 is still much higher than that of CCM, which denotes that the deformation of CCMC0.5 is more dominantly influenced by dislocations at the earlier deformation stage.

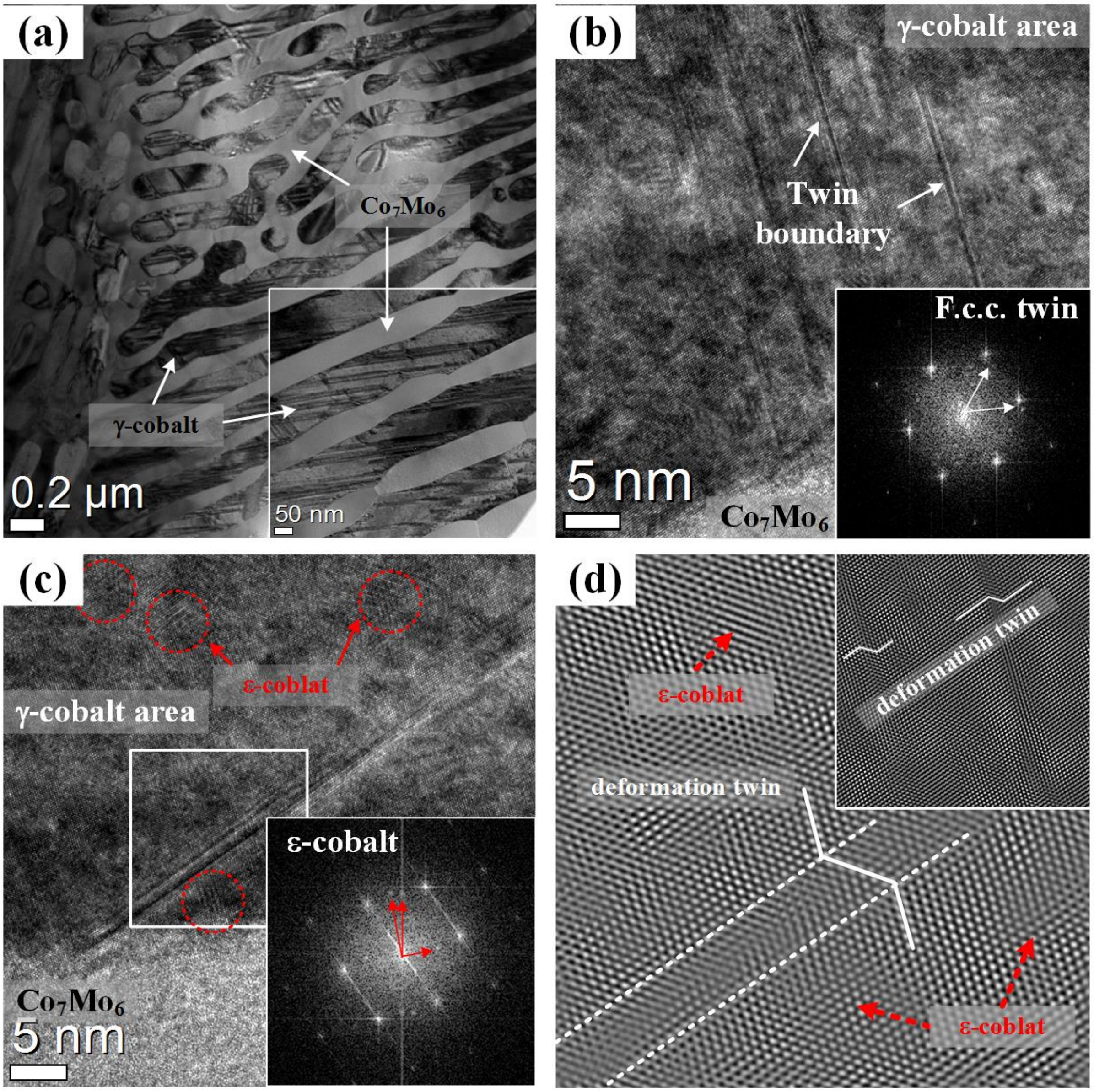

The supplementary Fig. 6 displays TEM images obtained from CCMC0.5 specimens deformed up to ~3 %. Slip bands are found at the γ-Co lamellae, as shown in supplementary Fig. 5 (a). The high magnification image and the fast Fourier transformation (FFT) pattern, shown in supplementary Figs. 6 (b) and (c), reveal the formation of deformation twins and ε-Co phase at the γ-Co lamellae, which are generated by a strain-induced phase transformation (SIPT) (see in Ref. 9-11). These ε-Co precipitates are located near the deformation twins situated at the γ-Co lamellae. The inverse FFT filtered image, obtained from the white squared area in supplementary Fig. 6 (c), clearly reveals the existence of deformation twins and ε-Co phase.

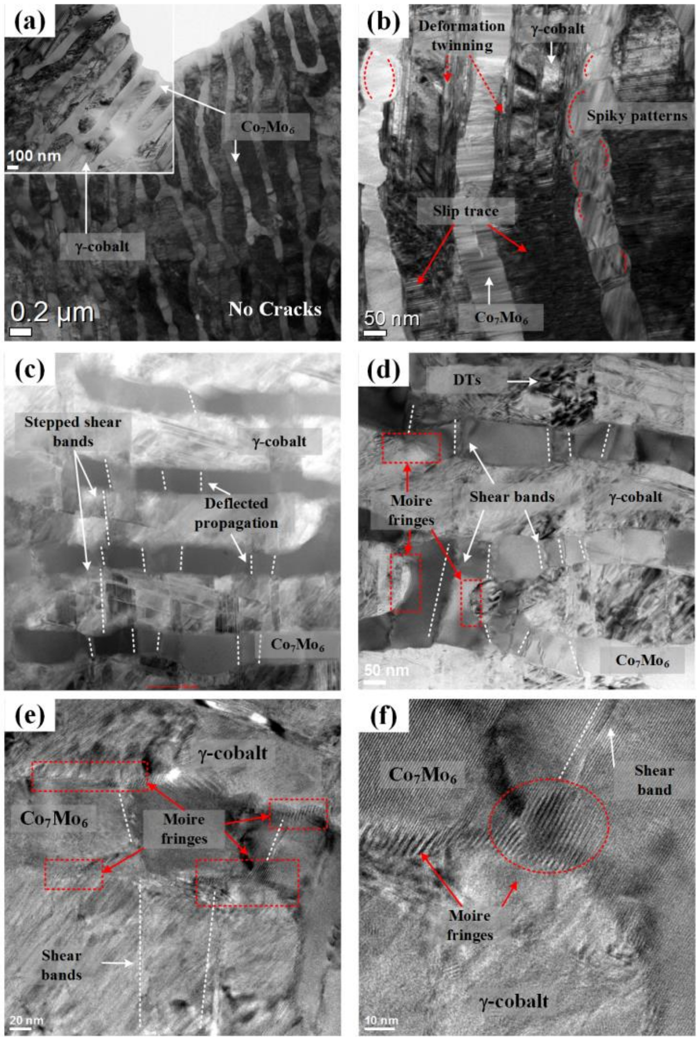

TEM bright field (BF) and high resolution (HR) images of a fractured CCMC0.5 specimen are shown in supplementary Fig. 7. Observation at low magnification reveals that a high density of deformation bands, giving evidence for dislocation-based deformation, are generated in both γ-Co and Co_7Mo_6 without crack generation at the lamellar interface, as shown by supplementary Fig. 7 (a). Moreover, the high magnification BF image in supplementary Fig. 7 (b) exhibits severely deformed γ-Co lamellae, showing a large amount of slip traces and deformation twinning. Furthermore, spiky morphologies are frequently found at the lamellar interface, as marked by dotted red lines. The supplementary Figs. 7 (c) and (d) display the presence of shear bands passing through the ultrafine lamellar interfaces. These shear bands are deflected in their propagation at the lamellar interface, which indicates interaction between shear bands and lamellar interface. Moreover, Moiré fringes are observed at the lamellar interface and the sharp edges of the spiky patterns are found at the tip of the shear band. The more highly magnified images [supplementary Figs. 7 (e) and (f)] clearly reveal that the shear bands are deflected at the lamellar interface and heavy Moiré fringes are located near the tip of the shear bands. This indicates that the lamellar interfaces in the CCMC0.5 alloy effectively accommodate the applied stress and then impede the propagation of deformation bands which involved in fracture immediately.

Supplementary table 1. Microstructural features and compressive mechanical properties of the as-cast $Co_{65-x}Cr_{13}Mo_{22}Cu_x$ alloys with x = 0, 0.5 and 1 at. %.

Alloys	Microstructure		Mechanical properties		
	V_f of γ-Co (%)	Average lamellar spacing (μm)	Yield strength (MPa)	Fracture strength (MPa)	Plastic strain (%)
CCM	50.3 ± 0.7	0.17 ± 0.05	2306.88 ± 3	2522.69 ± 3	1.31 ± 0.4
CCMC0.5	62.1 ± 0.8	0.11 ± 0.03	2300.30 ± 4	2753.96 ± 5	9.23 ± 0.6
CCMC1	67.5 ± 1.2	0.1 ± 0.02	2156.65 ± 5	2730.18 ±6	10.05 ± 0.5
